# One year later: mental health among employees in long-term care of the elderly after COVID-19 in Italy

**DOI:** 10.1192/j.eurpsy.2023.491

**Published:** 2023-07-19

**Authors:** C. Gesi, C. Grossi, E. Consorti, E. Vercesi, G. Cerveri

**Affiliations:** ^1^Mental health and addiction, ASST Fatebenefratelli-Sacco, Milan; ^2^Mental health and addiction, ASST Lodi, Lodi; ^3^Osservatorio ONDA, Milan, Italy

## Abstract

**Introduction:**

The COVID-19 emergency have imposed a great burden on the Italian health and social health system. In this context, healthcare workers (HCWs) have been exposed to high levels of stress. While many studies addressed the consequences of COVID-19 on hospital workers, little interest has been devoted to the employees of nursing homes.

**Objectives:**

To evaluate levels of depressive, anxious and post-traumatic symptoms in a population of nursing homes workers in Italy one year after the begin of the pandemic.

**Methods:**

The research involved 177 nursing homes, to evaluate the Mental Health outcome of the COVID-19 pandemic 12 months after the first lockdown on a large sample of workers. Participants answered a self-assessment tools aimed to assess the level of trauma experienced, the level of anxiety and depression, the quality of professional life and social and work adjustment.

**Results:**

A consistent level of psychological suffering in the HCWs 12 months after the first lock-down and after the third wave of Covid-19 is highlighted, in accordance with what has been observed in similar research. It turns out that about 30% of subjects, more often women, have elements suggestive of symptoms related to PTSD, with moderate levels of anxiety. On the other hand, 15% of the sample presents moderate levels of depressive symptoms and a severe impact on social and occupational functioning. Of these about 40% of staff has significant interference and just over 15% has a severe impact (see figure 1).

**Image:**

**Figure:**
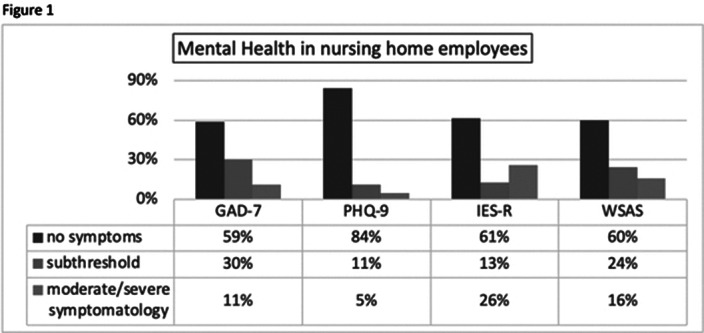

**Conclusions:**

Interventions tailored to support mental health are needed not only for HCWs from hospital units but also for those working in nursing homes and long-term care units.

**Disclosure of Interest:**

None Declared

